# Targeting the immune microenvironment for ovarian cancer therapy

**DOI:** 10.3389/fimmu.2023.1328651

**Published:** 2023-12-18

**Authors:** Felix Blanc-Durand, Lai Clemence Wei Xian, David S. P. Tan

**Affiliations:** ^1^ Department of Haematology-Oncology, National University Cancer Institute, Singapore (NCIS), National University Hospital, Singapore, Singapore; ^2^ Yong Loo Lin School of Medicine and Cancer Science Institute (CSI), National University of Singapore (NUS), Singapore, Singapore; ^3^ Yong Loo Lin School of Medicine, National University Centre for Cancer Research (N2CR) and Cancer Science Institute (CSI), National University of Singapore, Singapore, Singapore

**Keywords:** ovarian cancer, immunotherapy, immune microenvironment, tumor-infiltrating lymphocytes, tumor-associated macrophages, adoptive cell therapy, cancer vaccine

## Abstract

Ovarian cancer (OC) is an aggressive malignancy characterized by a complex immunosuppressive tumor microenvironment (TME). Immune checkpoint inhibitors have emerged as a breakthrough in cancer therapy by reactivating the antitumor immune response suppressed by tumor cells. However, in the case of OC, these inhibitors have failed to demonstrate significant improvements in patient outcomes, and existing biomarkers have not yet identified promising subgroups. Consequently, there remains a pressing need to understand the interplay between OC tumor cells and their surrounding microenvironment to develop effective immunotherapeutic approaches. This review aims to provide an overview of the OC TME and explore its potential as a therapeutic strategy. Tumor-infiltrating lymphocytes (TILs) are major actors in OC TME. Evidence has been accumulating regarding the spontaneous TILS response against OC antigens. Activated T-helpers secrete a wide range of inflammatory cytokines with a supportive action on cytotoxic T-cells. Simultaneously, mature B-cells are recruited and play a significant antitumor role through opsonization of target antigens and T-cell recruitment. Macrophages also form an important subset of innate immunity (M1-macrophages) while participating in the immune-stimulation context. Finally, OC has shown to engage a significant natural-killer-cells immune response, exerting direct cytotoxicity without prior sensitization. Despite this initial cytotoxicity, OC cells develop various strategies to induce an immune-tolerant state. To this end, multiple immunosuppressive molecules are secreted to impair cytotoxic cells, recruit regulatory cells, alter antigen presentation, and effectively evade immune response. Consequently, OC TME is predominantly infiltrated by immunosuppressive cells such as FOXP3^+^ regulatory T-cells, M2-polarized macrophages and myeloid-derived suppressor cells. Despite this strong immunosuppressive state, PD-1/PD-L1 inhibitors have failed to improve outcomes. Beyond PD-1/PD-L1, OC expresses multiple other immune checkpoints that contribute to immune evasion, and each representing potential immune targets. Novel immunotherapies are attempting to overcome the immunosuppressive state and induce specific immune responses using antibodies adoptive cell therapy or vaccines. Overall, the OC TME presents both opportunities and obstacles. Immunotherapeutic approaches continue to show promise, and next-generation inhibitors offer exciting opportunities. However, tailoring therapies to individual immune characteristics will be critical for the success of these treatments.

## 1 Introduction

Ovarian cancer (OC) is a leading cause of women mortality by cancer, with more than 200,000 death annually (1). Significant progress was made this last decade regarding the molecular characterization of OC, particularly the discovery of recurrent alterations in the homologous recombination (HR) pathway, such as BRCA1 and 2 mutations, among others. OC has served as a model demonstrating that inhibitors of the poly-ADP ribose polymerase (PARPi) are synthetically lethal in case of deficient HR (HRD) leading to a tremendous improvement in progression-free and overall survival (2–6). Approximately 40% of newly diagnosed OC cases are estimated to be, with half of them having BRCA mutations, making them potential beneficiaries of maintenance PARPi after a positive response to platinum-based chemotherapy (7, 8). However, despite these advancements, most HRD patients will eventually recur and effective strategies in the relapse setting are still lacking. Furthermore, for the majority of OC patients not classified as HRD, and those with no objective response to platinum, no targeted therapy has demonstrated a survival benefit highlighting the need for new effective treatment strategies.

The majority of OC cases are diagnosed at advanced stages (stage III/IV) and typically present with pelvic masses, peritoneal nodules and, less frequently, distant metastases (lymph nodes, lung and liver) (9). Classically, OC will spread and disseminates via malignant ascites composed of free-floating tumors cells or spheroids alongside with a complex ecosystem (10). The tumor cells, together with their surrounding non-malignant cells, extracellular matrix and various signaling molecules, create a unique tumor microenvironment (TME) that promotes cancer progression.

Over the past twenty years, targeting TME has become a key therapeutic strategy in solid tumors (11). The first successes involved Programmed-death 1(PD-1)/Programmed-Death ligand 1 (PD-L1) and cytotoxic T-lymphocyte-associated protein 4 (CTLA-4) pathways which are crucial mediators of cancer cells evasion from antitumoral T-cell-mediated cytotoxicity (12). Consequently, immune-checkpoint inhibitors (ICIs) targeting PD-1/PD-L1, and CTLA-4 were the first to be developed, demonstrating unprecedented benefits in selected patients and reshaping the therapeutic landscape for numerous cancers (13). Naturally, these ICIs were also tested on OC patients but ICIs as monotherapy or combined with chemotherapy have not been associated with any statistical significant survival benefits in phase III trials (14–17). It is suspected that the strongly immunosuppressive context and the number of actors involved in the OC TME are responsible for these disappointing results. Current data suggests that targeting the OC TME looks like an elusive goal, but it is also likely that focusing on T-cell activity and PD-1/PD-L1 pathway is too narrow in the context of the OC TME, and only an extensive characterization and more comprehensive understanding of the complex interaction between OC tumor cells and its microenvironment might change this paradigm.

## 2 Tumor-infiltrating lymphocytes in OC microenvironment

### 2.1 T-cells

**Figure 1:**
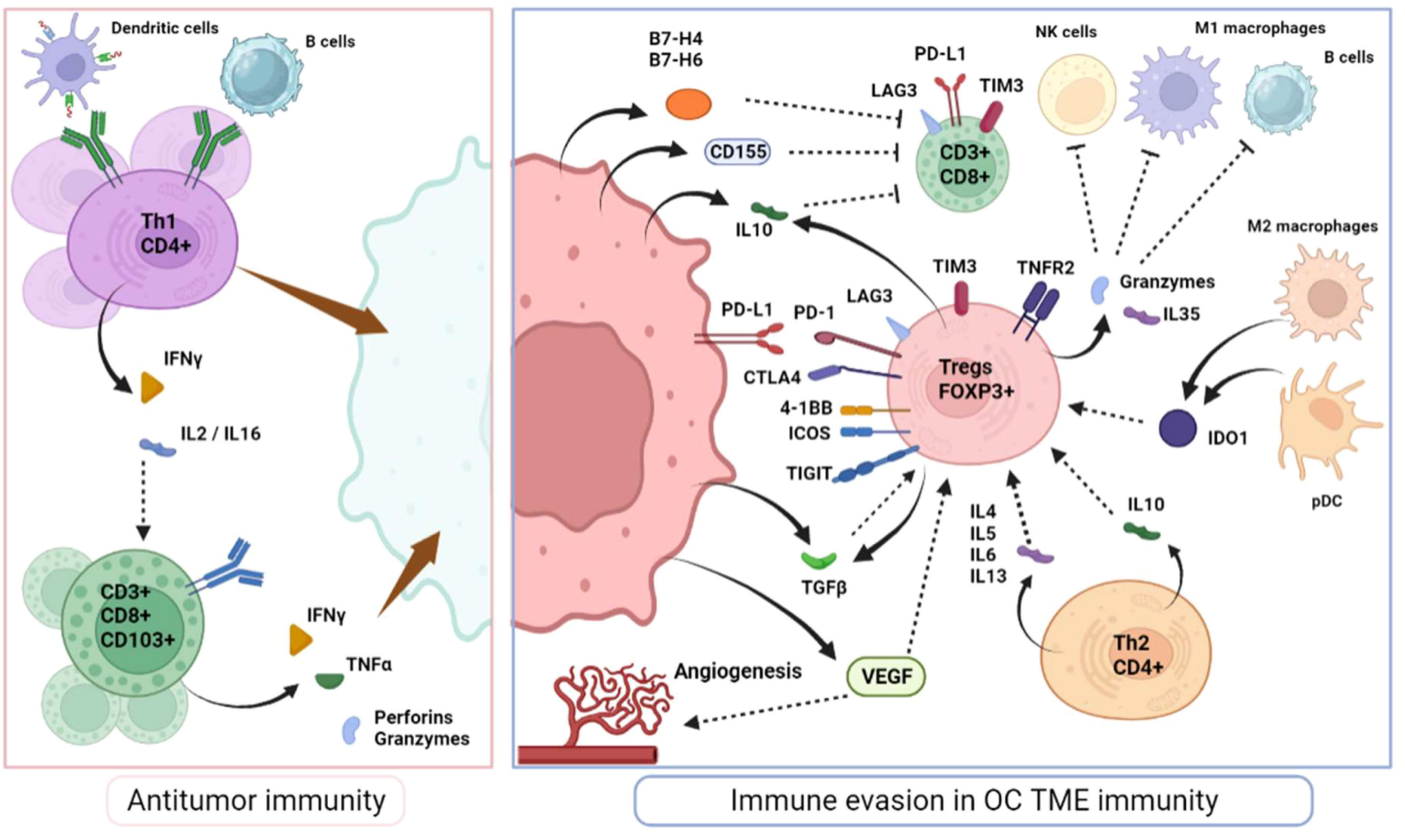
T cell immune response in OC microenvironnement.

Intraepithelial cytotoxic T-cells (usually defined as CD8^+^) recognize cancer-specific antigens carried by presenting cells and generate local inflammation via cytokine release, particularly interferon-gamma (IFNγ) and tumor necrosis factor alpha (TNFα) and recruitment of secondary immune actors that lead to the elimination of tumor cells (**Figure 1**). Therefore, they are a critical determinant of antitumor adaptative immunity. In OC, similar to many other solid tumors, a high infiltration of cytotoxic TILs appears to be a positive prognosis biomarker (18, 19). Importantly, this prognostic impact is particularly associated with TILs’ within the tumor epithelium rather than in stromal areas (20). However, both the abundance of CD8^+^ T-cell in primary tumors or metastatic localizations retain prognostic value (20). Among cytotoxic T-cells, it is well-established that several subpopulations coexist, exerting a broad range of inflammatory effects and supportive functions. CD44 and CD69 are typically associated with the initial activation of CD8^+^ T-cells. Subsequently, effector T-cells tend to express at their surface, the CD103 marker, a component of the alpha E/beta 7 integrin that binds to E-cadherin on epithelial cells, the killer cell lectin-like receptor G1 (KLRG1) and IL-2 receptor subunit-α (CD25) while downregulating L-selectin (CD62L), IL-7 receptor subunit-α (CD127) and CD27 (21, 22). CD103^+^ TILS have been linked to improved survival rates in OC patients (23, 24). Additionally, alternative effector CD8+ T-cells subsets (Tc2, Tc9 and Tc17 cells) display complementary inflammatory effects and mediate CD4+ T-helpers through specific interleukins secretion, particularly IL-4, IL-9 and IL-17, although their identification *in vivo* remains challenging (25–27).

CD4^+^ T-helpers (Th) are activated by antigen-presenting cells and have dualistic effects. On one hand, they provide cytokine support (Interleukine-2, IL-16 and IFNγ) to effectors cells like CD8^+^ T-cells and macrophages and trigger the recruitment of dendritic cells, consequently enhancing the duration of cytotoxic response (Th1 cells). Thus, most reports have observed a survival benefit associated with the number of CD4^+^ TILs in OC TME (28, 29). On the other hand, the Th2 profile drives the immunosuppressive context by the secretion of IL-4, 5, 6 and 10 and may explain why the prognostic value of CD4^+^ cells is inconsistent (30).

Based on the TCGA dataset and then confirmed on independent cohorts, HRD tumors (associated with a *BRCA1/2* mutation or not) exhibit a higher neoantigen load compared to HR proficient tumors (31). In addition, *BRCA1* mutations have been associated with a modest increase in TILs infiltration (32). However, this was not the case for *BRCA2* mutations (33).

Regulatory T-cells (Tregs) expressing FOXP3 transcription factors, are central actors in immune-regulation to avoid self-tissue toxicity (autoimmunity) but have a detrimental effect on the antitumor immune response (34). They can arise from naïve CD4^+^ exposed locally to transforming growth factor beta (TGFβ) and express elevated levels of CD25, or develop in the thymus and be recruited in the tumor TME (natural Tregs) (35). Tegs isolated from OC are highly activated as they tend to express multiple immunomodulatory molecules including PD-1, 4-1BB, TIGIT and ICOS and secrete important immunosuppressive factors such as IL-10, TGFβ and various granzymes (granzyme B, and perforins) that directly target other immune cells (36, 37). Indoleamine 2,3-dioxygenase (IDO1), vascular endothelial growth factor (VEGF), and B7-H4 are highly expressed in OC TME and have significant negative impact on effector T-cell proliferation while favoring Tregs proliferation and contributing to OC immune evasion (**Figure 1**) (38, 39). Moreover, a Treg subpopulation expressing the TNF receptor 1 (TNFR2), particularly immunosuppressive, is highly present in malignant ascites and could represent another factor driving OC dissemination (40).

Overall, increased Tregs infiltration (usually defined as CD4^+^/FOXP3^+^ phenotype) is correlated with immune tolerance and poor prognosis, including in OC (41–44). Consistently, a higher CD8/FOXP3 ratio appears to be correlated with improved outcomes (45, 46). Similarly, based on gene expression profiles, large retrospective studies have confirmed the large variability in TILs composition and the prognostic implications of specific immune infiltrates (47, 48). However, CD8^+^ T cells and Tregs were not found to be related with patients’ prognosis highlighting the probable coexistence of different functional subtypes not yet identified by this type of analyses (48).

Black plain arrows depict a secretion. Dotted stem arrows represent a inhibitory or a stimulatory signal. Tapered arrows represent a cytolysis effect.

### 2.2 B-cells

B-cells are classically known for their role in the antibody response against foreign antigens. As such, tumor-infiltrating B-cells stimulate antitumor immunity through opsonization of target antigens, complement activation and T-cell recruitment (49). However, recent studies have shown that they can also modulate immunity via the secretion of cytokine or other immunomodulatory molecules and through direct cell-cell interactions (50). Multiple B-cells subsets have been identified so far, including memory B-cells, antibody secreting B-cells (plasma cells), germinal center B-cells, regulatory B-cells (Bregs), etc. each with distinct surface markers. All of these subsets have been identified in OC, along with evidence of autoantibody response against OC antigens, such as P53 and NY-ESO-1 highlighting the importance of these immune cells in OC (51, 52).

Bregs are important immunosuppressive cells in the OC TME; they produce IL-10 and TGFβ, express several immune checkpoints at their surface (PD1, PDL1, OX40 etc) to impair CD4^+^ and CD8^+^ T cell proliferation and can promote tumor growth and progression via IL-35 signaling (53–55).

**Figure 2:**
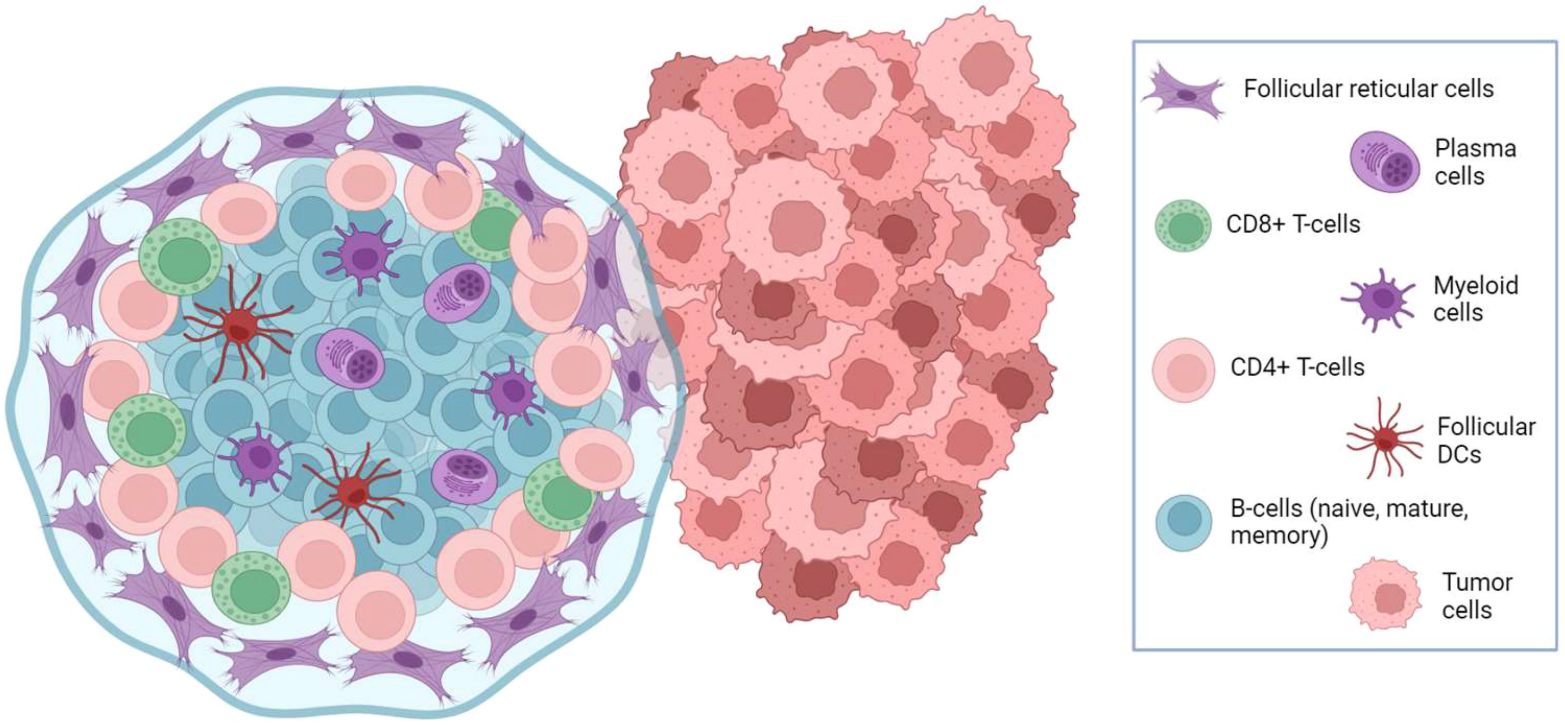
Schematic cellular composition of tertiary lymphoid structure in OC stroma.

Mature B-cells (CD20^+^) and antibody-secreting B-cells (CD19^+^) are reported to be associated with favorable outcomes in OC (56–58). Furthermore, tumor infiltration by plasma cells can participate in the recruitment of CD8^+^ and CD4^+^ T-cell and follicular dendritic cells in organized lymphoid aggregates inside tumor stroma (**Figure 2**), resembling lymph nodes architecture and called tertiary lymphoid structures (TLS). TLS are documented in around 30% of OC but tend to be associated with improved overall prognosis (59). Similarly, the coexistence of a high density of CD20^+^ B-cells with high levels of CD8^+^ and CD4^+^ T-cell is associated with favorable outcomes (57).

Conversely, some studies have suggested that high B-cell infiltration is associated with poor prognosis (60, 61). These discrepancies suggest that both the assessment of the absolute abundance of B-cells and the evaluation of the relative infiltration by different B-cells subtypes are necessary when addressing their clinical relevance. In addition, while the understanding of the importance of B cells in the antitumor immune response is rapidly growing, there is a clear need for better markers to precisely characterize B-cell infiltration.

### 2.3 NK cells

NK lymphocytes are a part of innate immunity and they exert direct cell toxicity using perforin and granzymes. They also produce various chemokines and inflammatory factors (IFNγ, TNFα, CCL5, XCL1, XCL2) against tumor cells without prior sensitization, mostly in an antigen-independent manner (62). NK activity is tightly regulated by the balance between inhibitory and stimulatory signals. They are classically activated by CD16, NKp30 and NKG2D, while inhibited by MHC proteins present at the surface of healthy cells (63). Additionally, they can influence B-cell and T-cell shaping as well as the selection of immature dendritic cells (DC) depending on the antigenic signal (64–66). Interestingly, the OC TME has shown to contain multiple NK inhibitory signals such as macrophage migration inhibitory factor (MIF) responsible for inhibiting NKG2D signal and B7-H6 ligand, which contributes to the downregulation of NKp30 signaling (67, 68).

In OC, NK cells often co-infiltrate tumor stroma along with CD8^+^ T-cells and are observed in high numbers in malignant ascites (24). Consequently, higher NK infiltration (CD56^+^) appears as a positive predictive biomarker (69). Conversely, some authors have observed the opposite effect when considering different markers for NK cell detection. For instance, higher CD16^+^ cells infiltration appears to be associated with poorer prognosis (61). Taken together, NK cells seem to be crucial components of the OC microenvironment. However, the lack of robust biomarkers to assess their relative abundance and potential impairment is a major limitation to existing data.

## 3 Myeloid precursors, dendritic cells and macrophages

### 3.1 Tumor-associated macrophages

Macrophages are a key component of innate immune response in humans and the most abundant immune cell within the TME (70). Macrophages residing in the TME are called TAMs and can originate from circulating monocytes recruited and matured locally, or from the differentiation of tissue-resident macrophages (71). They are programmed to participate in antitumoral immunity as M1-polarized macrophages, characterized by their phagocytic functions, defined as CD63^+^CD86^+^, and stimulated by IFNγ and granulocyte/macrophage colony-stimulation factor (GM-CSF). Their inflammatory activity involves various inflammatory molecules such as IL-1, IL-12 and IL-23, TNFα, and chemokine ligands (CXCL5, CXCL9 and CXCL12) as well as tumor-specific antigen presentation via major histocompatibility complex molecules (MHC class 1 and 2) (72, 73). In the context of OC, high M1-TAMs infiltration (along with a high M1/M2 ratio) constitute a good prognostic biomarker (74–76)

**Figure 3:**
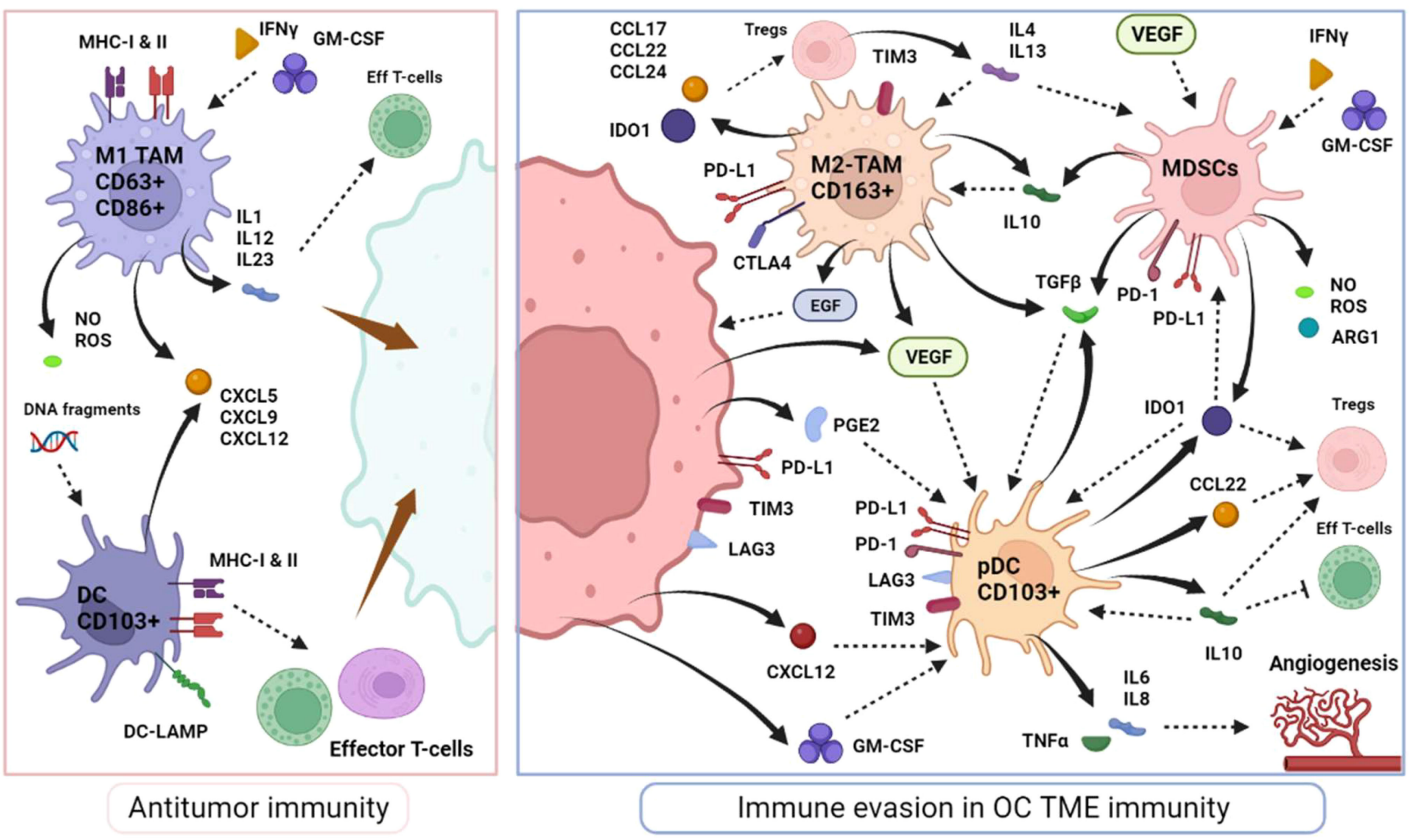
Myeloid cell immune response in OC microenvironnement.

On the other hand, it has been noted that M1-macrophages display strong plasticity and can transform into immune-suppressive protumoral immune cells as M2-polarized macrophages, under stimulation by prostaglandin E2 (PGE2), IL-4, IL-10 and IL-13 (77, 78). M2-macrophages express high levels of scavenger receptor class B (CD163) and secrete a variety of immunosuppressive molecules, such as IL-10, IL-13, TGFβ, CCL17, CCL22 and CCL24, which participate in monocytes differentiation to M2-macrophages (79, 80). They effectively modulate effector T-cell proliferation [through the expression of PD-L1 and CTLA4 receptors and arginase-1 (ARG1) activity] and enhance Tregs recruitment (mediated by CCL22) (81, 82). Aside from immunosuppression, M2 TAMs are involved in a wide range of actions including angiogenesis (VEGF secretion), TME remodeling and extracellular matrix degradation, adipocytes interaction and chemoresistance, all of which promote tumor progression and dissemination (83–88). In OC, M2-like TAMs play a role in the formation of spheroids during peritoneal dissemination via the secretion of epidermal growth (EGF) and VEGF and the absence of M2-like TAMs (as well as EGFR-blockage) seems to prevent metastatic dissemination in mouse models (**Figure 3**) (89, 90). Across various solid tumors, including ovarian cancer, a higher M2/M1-like ratio have shown to be correlated with advanced stage, high histologic grade, lymphovascular invasion and poorer outcomes (91, 92).

M2-like TAMs are the most represented immune cell in OC TME and play a pivotal role in OC carcinogenesis. Altogether, the interplay between OC and macrophages, and the subsequent balance between M1-inflammed macrophages versus M2-immunosuppressive cells, is likely critically important in the natural history of ovarian cancer.

### 3.2 Dendritic cells

Dendritic cells mainly home into lymph nodes or TLS and act as the most potent antigen-presenting cells. After capturing antigens, DCs mature into active DCs to present these foreign antigens on major histocompatibility complex, MHC-I and MHC-II, stimulating effector T-cells while providing inflammatory cytokines support (favoring Th1 immunity and cytotoxic T-cells proliferation) (93, 94). The abundance of mature DC (CD103^+^) appears to be correlated with the density of their secreted ligands such as CXCL9, CXCL10 and lysosomal-associated membrane protein 3 (DC-LAMP) and is associated with improved outcomes in OC (95–97). In OC, the abundance of DC-LAMP^+^ cells appears to be robustly associated with effective antitumor T-cell activity and better outcomes (96).

Nonetheless, in OC TME, tumor cells have shown to subvert normal physiological activity of DCs into an immature or an immunosuppressive phenotype, inhibiting T-cell activity instead of enhancing it. A subset of DC called plasmacytoid DCs (pDCs) is generally associated with inflammatory impairment. The immunosuppressive microenvironment, comprising CXL12, TGFβ, IL-10, VEGF, and IDO1, secreted by tumor cells (particularly in malignant ascites), Tregs and other immunosuppressive cells, drives the proliferation of immature and pDCS (98–100). In OC, immature DCs can secrete IL-6, IL-10, IDO1, ICOS-L and TGFβ among others, express PD-L1 and B7-H4 and thus, effectively disrupt the normal maturation of myeloid precursors, actively suppress T-cell responses, expand potent Tregs and consequently significantly weaken antitumor immunity (**Figure 3**) (101–105). In addition, pDCs are responsible for stimulating angiogenesis by the production of IL-8 and TNFα (98). Overall, most studies have revealed that the presence of pDCs correlates with low TILs infiltration, neoangiogenesis and, herein, is associated with poor prognosis (106, 107).

Therefore, the OC TME appears to profoundly alter DCs functions from potent inflammatory actors into central immunosuppressive mediators, fostering effective immune evasion and carcinogenic progression. The pivotal immunosuppressive role of DCs in the TME has led to considerable efforts in the development of DC-based vaccines intended to restore antigen-presenting capacity and improve antitumor immunity (108).

### 3.3 Myeloid-derived suppressor cells

Myeloid derived suppressor cells (MDSCs) are immature progenitors to myeloid cells such as dendritic cells and macrophages and they are stimulated by tumor cells to promote tumor growth and immunosuppression, particularly via inhibition of T-cells (109). The MDSCs are major producers of IL-10 in the TME, as well as a vast range of inflammatory mediators such as ARG1, nitric oxide (NO), reactive-oxygen species (ROS), TGFβ and IDO1, reinforcing their pivotal role in immune tolerance (110–112). In addition multiple factors highly expressed in the OC TME, have been shown to enhance MDSCs recruitment, such as cyclooxygenase-2 (COX2), prostaglandins, GM-CSF, and VEGF (**Figure 3**) (113–115). Other factors in the TME also tend to activate MDSCs, including IFNγ, IL-4 and TGFβ (111, 116, 117).

Active MDSCs inhibit effector CD4^+^ and CD8^+^ T-cells and attenuate NK-mediated cytotoxicity while also interfering with their migration capacity (118). Interestingly, OC favors their recruitment via inflammatory mediators like PGE2 which has been shown to induce the expression of PD-L1, CXCR4 and its ligand CXCL12 promoting angiogenesis, MDSCs accumulation and Tregs recruitment (119–121). Consistently, the OC TME contains high levels of tumor-infiltrating MDSCs, and an elevated number of MDSCs is associated with advanced stages, high-grade tumors, and poor prognosis (120, 122–124).

Nonetheless, the lack of specific markers has hampered their precise characterization and the understanding of their impact on immunosuppression

Black plain arrows depict a secretion. Dotted stem arrows represent a inhibitory or a stimulatory signal. Tapered arrows represent a cytolysis effect.

## 4 Compositional heterogeneity within the OC TME

The understanding of the composition and cross-talk between TME components remain critical in decoding the complex workings of the TME. Conventional bulk transcriptome analysis has made substantial contributions to our comprehension of the TME components. Bioinformatic deconvolution methods, such as CIBERSORTx and xCell, enable the inference of multiple cell types from bulk RNA-seq data, thus unveiling the heterogeneity of the TME. These methods employ predetermined gene signatures that represent each cell type, providing a general estimate of cell type abundance based on bulk data (125, 126). Immune deconvolution of TCGA data defined three immune subtypes: immune-activated, immune-suppressed and immune-desert, characterized by differing abundances of CD8^+^ T cells, M1 and M2 macrophages, and exhibiting distinct survival trends (127). Notably, neutrophil infiltration was associated with poor survival outcomes in only two out of the four molecular subtypes identified in HGSOC (128), underscoring the necessity to understand the tumor context and its surrounding components in order to elucidate the various immune-related influences on survival.

The emergence of single-cell RNA sequencing (scRNA-seq) technology has empowered us to explore the transcriptomic diversity of tumors and microenvironment components with unprecedented resolution (129). By incorporating unique molecular identifiers (UMIs) in droplet-based protocols, scRNA-seq allows the simultaneous analysis of thousands of individual cells from a single biopsy. This facilitates the detection of small cell populations that may hold prognostic significance. Several scRNA-seq studies have characterized components of OC TME, revealing extensive subpopulations of tumor, stromal and immune cells. For instance, scRNA-seq profiling of 12 HGSOC biopsies obtained from various sites (ovarian, peritoneal and omentum) revealed the existence of 32 stromal subclusters. Among these, distinct functional phenotypes of mesothelial cells, endothelial cells and fibroblasts were identified, with several showing correlation with poor survival. In another study focused on the landscape of infiltrating T cells in seven cases of HGSOC, 22 subclusters of T cells were detected, comprising 7 clusters of CD4^+^ and 15 clusters of CD8^+^ cells, each characterized by their unique signature genes (130). This discovery underscores the inherent functional differences within the T cell compartment.

Significantly, distinct TME niches can coexist within the same patient. Profiling OC at different tumor sites has revealed compositionally diverse TMEs within individual patients. In a study involving multi-site biopsies from 42 HGSOC patients. Comparative analysis revealed an enrichment of dysfunctional T cell in adnexal sites compared to non-adnexal sites (131). Similarly, the myeloid and DCs compartment exhibited significant compositional variations between solid tumor foci and ascites, both within and among patients. These findings illustrate the existence of site-specific immunophenotypes, that may impact the response to current treatment modalities. Differences in immune context across various tumor sites may thus contribute to the limited efficacy of current immunotherapy regimens. Approaches potentially effective for ovarian tumors may not necessarily apply as effectively to omental localizations and vice versa.

## 5 Spatial influences in TME

In addition to compositional differences in the TME, the classification of ovarian tumors into “immune-hot”, “immune-desert” or “immune-excluded” categories can be stratified based on the histological localization of CD8^+^ T cells. “Immune-hot” tumors are characterized by the presence and high density of CD8^+^ T cells within the tumor bed. “Immune-desert” tumors are lack CD8^+^ T cells in both the tumor bed and the tumor edges. Lastly, “Immune-excluded” tumors are defined by the retention of CD8^+^ T cells at the tumor edges without entering the tumor islets. Similarly, considering stromal CAFs known to regulate tumor immunity, the spatial positioning of CAFs also holds significance in mediating immunosuppression in cancer. In lung cancer, the histological presentation of alpha-smooth muscle actin (αSMA) CAFs lining tumor aggregates instead of being spread across the stroma is associated with decreased T cell infiltration into tumor nests, indicating a more “immune-excluded” phenotype. These observation underscore the importance of cell localization in mediating different states of the TME (132). Indeed, mounting evidence across various cancer types suggests that the spatial positioning of the cellular components within the TME, in relation to tumor cells, immune compartments, and vasculature, plays a crucial role in regulating anti- or pro-tumoral responses and understanding intricate intercellular networks (133–135).

Our current understanding of the OC TME has been primarily shaped by traditional methods of immune deconvolution, such as immunohistochemistry (IHC), and hematoxylin and eosin (H&E) tissue staining. However, these low-content methods have limitations in their ability to comprehensively unravel the spatial architecture of the TME, which hinders the discovery of novel distributions of cell populations. Advanced spatial techniques include transcriptomic-based approaches such as Nanostring GeoMx (136) and 10X Visium, and protein-based methods, including OPAL-based multiplexed fluorescent imaging (137) and imaging mass cytometry (138). A key advantage of these techniques allows for the detection of multiple genes or proteins of interest within the spatial architecture, which were restricted in conventional IHC or *in-situ* hybridization transcriptomic methods. **Table 1** highlights some of these spatial platforms in research.

**Table 1:** Overview and applications of spatial technologies.

Spatial Transcriptomics (RNA-based)						
Technique	Vendor	Resolution	Tissue Type	Number of RNA targets per tissue section (plexing)	General Applications	References to OC-related studies
GeoMX Digital Spatial Profiling (DSP)	Nanostring	Cell-type	FF/FFPE	Whole Transcriptome (~18000+)	Interrogate the biology of cell types of interest and discovery of biomarkers correlating with immune infiltration status and survival prognosis.	(139, 140)
Visium	10X Genomics	Near cellular (1-10 cells per spot; 55uM)	FF/FFPE	Whole Transcriptome (~18000+)		(141, 142)
Slide-Seq	Curio Biosciences	Near cellular (10uM)	FF	Whole Transcriptome		NIL
Stereo-Seq	Beijing Genomic Institute	Nano-cellular	FF/FFPE	Whole Transcriptome		NIL
Spatial Proteomics						
**Technique**	**Vendor**	**Resolution**	**Tissue Type**	**Number of Protein targets per tissue section (plexing)**	**General Applications**	**References to OC-related studies**
OPAL-based multiplexed fluorescent imaging	Akoya Biosciences	Cellular	FF/FFPE	Up to 9	Identification of cellular neighborhoods and spatially restricted cell types in relation to survival.	(143, 144)
Imaging Mass Cytometry	Standard Biotools	Cellular	FF/FFPE	Up to 50		(138)
MIBI	Ionpath	Subcellular	FF/FFPE	Up to 100		(145)
PhenoCycler-Fusion	Akoya Biosciences	Subcellular	FF/FFPE	Up to 100		NIL

FF, Fresh Frozen; FFPE, Formalin-fixed paraffin-embedded.

These techniques are conducted on fresh frozen or FFPE tissues, preserving the tissue architecture and cellular morphology. This allows for information to be obtained from intact archival tissue in the original physiological context. The simultaneous detection of multiple genes or proteins in a single-tissue section enables the in-depth exploration of the biology of relevant cell types and their phenotypes within the TME, reaching unprecedented levels of detail (146). For example, application of imaging mass cytometry (IMC) on HGSOC tissues, allowed for the detection of 21 proteins of interest simultaneously in a single tissue section enabling profound levels of cell phenotyping, and capable of distinguishing functional differences within the same cell type, such as the detection of M1- from M2-polarized macrophages (138). The greater number of targets detected generally unveils the TME composition beyond the capabilities of traditional IHC modalities which detects only one single marker in a tissue sample. In addition, the preservation of spatial information facilitates the identification of unique spatial patterns of immune cells within the TME. It is established that cells closely clustered in proximity are likely to engage in stronger and more frequent interactions, potentially leading to functional remodeling of the TME and influencing treatment responses. These spatial patterns enable the identification of specific cellular neighborhoods and cell-cell crosstalk with distinct anti-tumor characteristics. For instance, multiplexed imaging analysis of HGSOC tissues revealed a prognostically relevant proliferative Ki67^+^ tumor cell population associated with enhanced spatial tumor-immune interactions involving cytotoxic and helper T cells in BRCA-mutated tumors, correlating with improved survival. Interestingly, the prognostic role of CD8^+^ T cells was conveyed only by their spatial arrangement near Ki67^+^ tumor cells rather than their relative abundance (147). In the TOPACIO trial (148) where OC patients received a PARP inhibitor (niraparib) combined with an anti-PD-1 antibody (pembrolizumab), spatial analysis of TME cellular neighborhoods revealed spatial clustering of exhausted CD8^+^ T cells with PDL1^+^ macrophages as a central factor in driving response towards PD1/PDL1-based immunotherapy (149). In a study examining CAFs in short-term versus long-term OC survivors, the APOE(CAFs) - LRP5(tumor) interaction pattern between CAFs and tumor cells at the tumor-stroma interface was associated with short-term survival. This specific cell-cell interaction may play a crucial role in modulating the malignant phenotype of OC, potentially serving as a spatially resolved prognostic biomarker for patient survival.

Overall, spatial features within the TME, including the spatial interactions of immune cell subpopulations, have the potential to be linked to treatment response and hold promise for more effective immunotherapeutic strategies and patient stratification in OC.

## 6 Non-cellular immune evasion actors

### 6.1 Immune-checkpoints

PD-L1 is a coregulatory molecule secreted on the surface of multiple immune cells and cancer cells. When it binds to its receptor PD-1, mainly present on lymphocytes, it generates an inhibitory signal (150). Importantly, PD-L1 expression on TILS can be induced by various factors secreted by TILS themselves or NK cells, such as IFNγ (151, 152). OC infiltrating DCs and MDSCs can also express both PD-1 and PD-L1 on their surface, promoting T-cell exhaustion (105, 121). However, there are inconsistent results regarding the expression of PD-L1 in OC, as each report uses different scoring system, different positivity thresholds, and different antibodies. It is generally accepted that around 30% of ovarian cancer cells show significant (≥5%) PD-L1 expression, whereas a higher rate of cases present concomitant PD-L1 on TILS (153–155). As for infiltrating lymphocytes, no significant association between PD-L1 expression and *BRCA1/2* mutations has been observed (154, 156). Paradoxically, high PD-L1 expression is generally associated with improved outcomes, in particular in case of high number of CD8^+^ TILS (153, 157–159). Nonetheless, these results may simply suggest that PD-L1 expression behaves as a surrogate marker of CD8^+^ TILS infiltration.

Cytotoxic T-lymphocyte-associated protein 4 (CTLA-4) is another transmembrane receptor, belonging to the CD28 family, expressed on the T-cell surface that binds to CD80/CD86 (B7 ligand) to transmit an inhibitory signal. Upon T-cell activation, CTLA-4 is released to the cell surface and regulates effector T-cell and Tregs proliferation, B-cell response, and indirectly IL-2 production (160, 161). Physiologically, CTLA-4 exerts an inhibitory action on both effector and regulatory immune cells to avoid uncontrolled inflammation. Consequently, CTLA-4 inhibitors have been developed to promote T-cell expansion and increase antitumor response. In the context of OC, significant heterogeneity exists between CTLA-4 expression, suggesting different levels of B7-ligand pathway inhibition (162). Herein, it is anticipated that only the subset of patients with high CTLA-4 expression will benefit from CTLA-4 specific inhibition.

Likewise, lymphocyte activating 3 (LAG3) is also an coinhibitory checkpoint expressed on various mature immune cells, including activated CD4^+^ and CD8^+^ T-cells, FOXP3^+^ Tregs, NK cells and DCs (163–166). The activated LAG3 signal (induced by MHC class-II molecules) acts synergistically with PD-L1 signaling in disrupting CD4^+^ and CD8^+^ functions, inducing their apoptosis while favoring Tregs proliferation (167). Its expression has been found to be associated with increased PD-L1 expression and a higher number of CD8^+^ T-cells in OC TME (154, 168). Around 10% of TILS infiltrating the OC TME express LAG3 without any significant association with tumor mutational profile or patient outcomes (156, 169).

T cell immunoglobulin and mucin domain containing protein 3 (TIM3), also known as hepatitis A virus cellular receptor 2, is an immune checkpoint receptor present on the surface of dysfunctional CD4^+^ and CD8^+^ T-cells, Tregs, NK-cells, M2-macrophages and DCs (170–172). On T-cells, it binds to galectin-9 and carcinoembryonic antigen-related cell adhesion molecule-1 (CEACAM1) carrying both an inhibitory signal inducing effector T-cell exhaustion on one hand, and a stimulatory signal enhancing Tregs immunosuppressive state on the other hand (173). At the same time, TIM3 is constitutively expressed on DCs and macrophages and appears to favor their immunosuppressive functions within the TME, although this mechanism is still unclear (174, 175). Like LAG3 and PD-L1, the expression of TIM3 is significantly correlated with TILS in OC (176). Importantly, TIM3 expression on OC TILS is very frequent, and most TIM3^+^ cells coexpress one or multiple other coregulators simultaneously (156, 177).

Platinum remains the cornerstone in the treatment of OC and is associated with compelling activity in 1^st^ line, with response rates of more than 70% (178). Consequently, platinum chemotherapy has been increasingly used in the neoadjuvant chemotherapy (NACT) setting to decrease tumor burden and help achieve completeness in surgery (179). This window-of-opportunity between diagnostic biopsies and interval debulking surgery has allowed the comparative assessment of OC immunogenicity before and after chemotherapy exposure. Interestingly, multiple teams have demonstrated immunogenic cell death with a positive impact of NACT on CD4^+^, CD8^+^ and NK-cells, suggesting an antitumor immune response induced by the chemotherapy (46, 180–182). Conversely, some reports have also observed a significant increase in PD-L1, CTLA4, and LAG3 expression under NACT which probably tempers the immunogenicity (156, 182–184). Altogether, these observations suggest that NACT primes an immunocompetent TME in which ICIs could be more efficient.

### 6.2 Cytokines, VEGF and interleukins

IDO1 is an immune regulatory factor induced by the inflammation cascade, including signaling by interferons, IL-10 and TGFβ). It is often secreted by cancer cells, DCs and macrophages, and rarely by TILS, and its expression generates an immune-permissive TME (185). IDO1 plays a crucial role in activating Tregs, allowing a stable and IDO1-independent immunosuppressive TME (186). Additionally, IDO1 appears to upregulate PD-L1 and CTLA-4 expression, indirectly affecting T-cell activation. Furthermore, IDO1 also contributes to the recruitment of MDSCs and enhances their suppressor functions (187, 188). In OC, IDO1 is frequently expressed, and higher expression is associated with chemoresistance and poor prognosis (189).

VEGF is a family of proteins regulated by hypoxia-induced genes (HIF) and EGF, and its role in OC angiogenesis, progression and intraperitoneal dissemination is well-established (190, 191). VEGF has also been shown to induce the evasion of innate and adoptive immune response by inhibiting the maturation of DCs and promoting Tregs activity. In the context of OC, a large amount of VEGF is secreted in the TME, particularly in malignant ascites, in contrast to normal ovarian tissue and non-malignant ascites (192, 193). Recently, it has been observed that VEGF expression can be induced by multiples factors present in OC TME, such as PGE2, TGFβ, and TNFα, reinforcing its close relationship with the immunosuppressive context (194, 195). Elevated levels of VEGF are associated with poor prognosis in OC patients (99, 196, 197). Based on these considerations, bevacizumab, a VEGF inhibitor, has been intensively evaluated in OC patients and has demonstrated higher response rates and improved outcomes. It is now FDA-approved and routinely prescribed (198, 199). Interestingly, bevacizumab has been shown to significantly increase T-cells and B-cells infiltration, enhance DC maturation and block Tregs proliferation in colorectal and breast models (200–202). Altogether, these reports suggest that using VEGF-targeting agents may give positive effects on the OC TME and enhance antitumor immune responses.

As previously mentioned, the cytokine context within the OC TME, plays a crucial role in creating an immunosuppressive state that allows tumor progression and immune evasion. IL-10 produced by various immune cells and OC cells themselves, is a soluble factor which acts as a potent negative regulator of antitumor inflammation. Its precise mechanisms on effector immune cells are not fully understood, but it likely involves the induction of other inhibitory factors like PGE2, the active blockage of DC maturation, the induction of PD-1 expression and the deletion of antigen presentation by ovarian tumor cells (203, 204). Interestingly, PD-1 blockade can lead to a high release of IL-10 in OC TME, suggesting a compensatory mechanism to escape inflammatory response (204). Similarly, IL-6 produced mainly by macrophages and cancer cells, plays a critical role in promoting angiogenesis, ascites development, and T-cell exhaustion (205, 206). Overall, both IL-10 and IL-6 are independent predictors of poorer outcomes in OC (206–209).

TGFβ is another cytokine that is highly expressed in OC TME and has a dual role, acting as both a tumor suppressor and a promotor of tumor progression and immunosuppression. Cancer cells that escape the immunosuppressive effects of TGFβ can overexpress it to promote invasiveness, neoangiogenesis and immune evasion (210, 211). TGFβ directly affects the activity of T-cells, NK-cells and DCs and can regulate the release of inflammatory cytokines (212).

The OC TME is deeply influenced by numerous other cytokines as well, with each having a different impact on progression and immune activity. Understanding the roles of these cytokines and their interactions in the TME is crucial for identifying potential targets to overcome immunoresistance and improve therapeutic outcomes.

Within the intricate landscape of the OC TME, non-immune elements have emerged as integral contributors to immune evasion, offering insights into potential therapeutic strategies. Cancer-associated fibroblasts (CAFs), activated by TGF-β signaling and governed by aberrant signaling pathways, have been identified as key orchestrators of immunosuppression. They promote extracellular matrix remodeling, notably collagen engineering, that physically hinder immune cell infiltration (213). Furthermore, CAFs engage metabolic reprogramming, fostering a nutrient-depleted TME unfavorable for effector T cell function (144). Endothelial cells within the tumor vasculature also play a pivotal role, participating in immune evasion by regulating immune cell trafficking and immune checkpoint molecule expression (214). Notably, autotaxin, an enzyme produced by various cell types in the TME, generates lysophosphatidic acid (LPA), which can enhance tumor growth and impair immune cell function through LPA receptor signaling (215). Collectively, these non-immune elements underscore once again the complexity of immune evasion mechanisms while representing potential targets for therapeutic interventions (216, 217)

## 7 TME particularities of rare epithelial OC subtypes

### 7.1 Clear cell ovarian cancer

Clear-cell ovarian cancer (OCCC) represents the second most frequent subtype of epithelial ovarian cancer, comprising approximately 15% of cases (218). Unlike high-grade serous OC, OCCC is often associated with endometriosis, and is typically diagnosed in younger women. It is also characterized by a tendency to present as localized diseases (219, 220). Molecularly, OCCC displays distinct profiles compared to other subtypes, with high rates of *ARID1A* loss-of-function and *PIK3CA* activating mutations, while *P53* loss-of-function mutations are observed in only 20% of cases (221, 222). These molecular characteristics suggest the presence of a strong immunosuppressive microenvironment. The PI3K/AKT/mTOR pathway is reported to promote immune evasion by increasing resistance to cytotoxic T-cell induced apoptosis, reinforcing Tregs functionality and escaping death receptor signals via PD-L1 and LAG3 expression (223–226). Furthermore, endometriosis and ARID1A mutations also contribute independently to the immunosuppressive environment. they have been shown to reduce T-cell infiltration and enrich the TME with PD-L1 expression and various other immunosuppressive cytokines, including IL2, IL6, IL10, TGFβ and TNFα (227–231). Interestingly, recent studies reported that coexistent *PIK3CA*/*ARID1A* mutations in OCCC are responsible for high levels of IL-6 and this mechanism may promote cancer growth and as such, could represent a potential therapeutic target in OCCC (232). Overall, OCCC appears to be the least infiltrated OC subtype by CD8^+^ TILS and, contrary to the others subtypes, it has been suggested to have a negative impact on survival (44, 231, 233).

### 7.2 Mucinous ovarian cancer

Mucinous OC (MOC) is known to have a poor response to platinum-based chemotherapy and advanced diseases are associated with a dismal prognosis (234). Molecularly, MOC often carries KRAS mutations, which are commonly associated with P53 mutations and ERBB2 amplifications, resembling the molecular background of colorectal carcinomas (235). One interesting aspect of MOC is that around 20% of cases exhibit mismatch repair deficiency, leading to a very high mutational load, increased neoantigens levels and potential sensitivity to immune checkpoint inhibitors (236). However, the immune context of MOC is poorly understood and limited data are available. In this regard, a recent study by Meagher et al, investigated the TME of MOC in 23 cases and reported high heterogeneity across these tumors. They observed that advanced stages had a higher number of M2-like macrophages and a higher density of FOXP3 Tregs in the tumor stroma was associated to poorer prognosis. Overall, MOC were not found to have an immune competent TME as most of the cases were classified as “cold” tumors based on T-cell infiltration and PD-L1 expression, which may explain its unfavorable prognosis and suggests resistance to immunotherapy even though there is no published clinical data supporting this assumption (237).

### 7.3 Low-grade ovarian cancer

Low-grade OC (LGOC) is a relatively rare epithelial OC subtype, accounting for less than 10% of. It typically occurs in younger women and is often diagnosed at an advanced stage. Compared to high-grade serous OC, LGOC is generally associated with more favorable outcomes (238). Their molecular background is characterized by a low mutational burden and recurrent mutations in the RAS/RAF pathway (239). However, little is known regarding about the immune infiltrate in LGOC. Studies have reported that LGOC tend to have fewer TILS and a lower number of CD68^+^ TAMs compared to high-grade tumors (240, 241). In addition, the TAMs associated with LGOC appear to be less polarized into M2-like macrophages (lower CD163/CD68 ratio) compared to HGOC (241). Taken together, given the low immune infiltration in LGOC, it is thought that these tumors may not be ideal candidates for immunotherapies.

## 8 Therapeutic implications

### 8.1 Targeting immune-checkpoint inhibitors

The principle behind targeting immune checkpoints is to block the natural inhibitory pathway that cancer cells use to suppress the activity of T cells, thereby allowing the immune system to maintain an effective immune response. The first class of ICI approved were PD-1/PD-L1 inhibitors, which block the interaction between PD-1 receptor on T-cells and the PD-L1 ligand, enabling T cells to remain active and attack cancer cells (150). In OC, PD-1/PD-L1 inhibitors monotherapy has shown limited activity, with an objective response rate (ORR) of approximately 10% and disease stabilization in around 30% of cases (16, 242, 243). To improve activity, considering the strong rationale of immunogenic cell death induced by cytotoxic agents and the close relationship between angiogenesis and TME, investigators have explored combinations strategies with chemotherapy and bevacizumab. However, large phase 3 trials have failed to demonstrated any benefit of PD-1/PD-L1 inhibitor combinations (**Table 2**) (14–17).

**Table 2:** Summary of Key Trials Related to PD-1/PD-L1 Inhibitors in Ovarian Cancer.

Study	Design	Population	Experimental arm	ORR (%)	PFS (months)	OS (months)	Reference
**KEYNOTE-100**	Open-label, single arm, phase II trial	PROC N=376	Pembrolizumab	8.0	2.1	17.6	(242)
**JAVELIN 100**	Randomized, open-label, phase III trial	Treatment naïve OC N=998	CPT Maintenance Avelumab	30	16.8 (HR=1.43)	NE	(17)
			CPT + avelumab Maintenance Avelumab	36	18.1 (HR=1.14)	NE	
**JAVELIN 200**	Randomized, open-label, phase III trial	PROC N=566	Avelumab	4	1.9 (HR=1.68)	11.8 (HR=1.14)	(16)
			Avelumab + PLD	13	3.7 (HR=0.78)	15.7 (HR=0.89)	
**IMagyn050**	Randomized, double-blind, phase III trial	Treatment naïve OC N=1301	CPT + bevacizumab + atezolizumab	NA	19.5 (HR=0.92) PDL1 + 20.8 (HR=0.80)	NE PDL1^+^ NE	(15)
**ATALANTE**	Randomized, double-blind, phase III trial	PSROC <2 prior lines N=1301	Carboplatin doublet + bevacizumab + atezolizumab	NA	13.5 (HR=0.83) PDL1 + 15.2 (HR=0.86)	35.5 (HR=0.81)	(14)
**NINJA**	Randomized, open-label, phase III trial	PROC N=363	Nivolumab	7.6	2.0 (HR=1.5)	10.1 (HR=1.03)	(244)
**NRG GY003**	Randomized, open-label, phase II trial	Relapsed OC N=100	Nivolumab + Ipilimumab	6-month ORR 31.4	3.9 (HR=0.53)	28.1 (HR=0.79)	(245)
**MEDIOLA**	Open label Phase II trial	PSROC Somatic BRCA mutation N=66	Olaparib + Durvalumab Olaparib + Durvalumab + Bevacizumab	31.3	5.5	26.1	(246, 247)
				77.4	14.7	31.9	
**TOPACIO**	Open label, Phase I/II trial	Relapsed OC N=62	Niraparib + Pembrolizumab	18	3.4		(148)
**DUO-O**	Randomized, double-blind, phase III trial	Treatment naïve OC N=1104	Carboplatin doublet + bevacizumab + durvalumab + placebo	NA	20.6		(248)
			Carboplatin doublet + bevacizumab + durvalumab + olaparib	NA	24.2		
**MOCCA**	Randomized, open-label, phase II trial	Relapsed OCCC <4 prior lines	Durvalumab	10.7	1.9	NA	(249)
**PEACOCC**	Open-label, single arm, phase II trial	Relapsed OCCC ≥1 prior line	Pembrolizumab	25	3.1	17.8	(250)

NA, Not Reached.

Despite the challenges in identifying reliable biomarkers for predicting response to ICI, some studies have shown that patients with higher PD-L1 expression (based on CPS score) may benefit more from adding PD-1/PD-L1 inhibitors to the standard treatment regimen. In the IMAGYN050 trial, patients with ≥5% expression of PD-L1 on the immune cells, or patients with ≥1% positive tumor cells, seemed to benefit from adding atezolizumab (PD-L1 inhibitor) to chemotherapy with bevacizumab (HR=0.64 [0.43-0.96] and HR=0.41 [0.19-0.90]) (15). However, these corresponded to only 20% and 6% of the overall population. Conversely, in the JAVELIN 200 and ATALANTE trials, PD-L1 positive patients did not benefit from adding ICI nor did the CD8^+^/PD-L1^+^ positive subgroup (14, 16). Interestingly, in some trials, patients with OCCC appeared to benefit more from ICI therapy. Consequently, dedicated trials have tested ICI in this population and reported encouraging responses with 11-25% ORR and up to 38.5% when associated with bevacizumab (249–251). Disappointingly, the only randomized trial comparing single-agent chemotherapy to durvalumab (PD-1 inhibitor) in OCCC patients, did not show improved outcomes in the ICI arm, highlighting the need for better biomarkers (249).

The complexity of the OC TME, including the multiplicity of immunomodulators, is most likely the reason for these contradictory results. To overcome these challenges, next-generation trials are exploring new targets, including other immunoregulatory checkpoints more suitable for the OC TME. Combinations of a PD-1 inhibitors with CTLA-4 inhibitors (nivolumab+ipilimumab) have demonstrated increased activity compared to monotherapy with a 31.4% ORR, versus 12.2% with nivolumab alone, at the cost of frequent severe adverse events (245). Likewise, various large ongoing trials are evaluating the benefits of adding a CTLA-4 inhibitor to a PD-1/PD-L1 inhibitor plus chemotherapy. Furthermore, clinical trials with ICI targeting novel checkpoints like TIM3 and LAG3, are being tested in OC, and the results are eagerly awaited (NCT02608268, NCT03099109, NCT04611126, NCT03538028, NCT03365791).

PROC: platinum-resistant OC; PSROC: platinum-sensitive recurrent OC; CPT: Carboplatin+Paclitaxel; PLD: Pegylated liposomal doxorubicine; OCCC: clear-cell ovarian cancerAs previously mentioned, HRD tumors tend to present higher levels of TILS and neoantigens load, suggesting a potential combination strategy of ICI with PARP inhibitors (31, 32). Additionally, PARP inhibitors have shown to induce immunogenic cell death through the activation of the cytosolic DNA sensor cyclic GMP-AMP synthetase (cGAS) and stimulator of interferon genes (STING) pathway (252), further enhancing the potential synergy between PARP inhibition and immunotherapy. The MEDIOLA trial, which tested the combination of olaparib and durvalumab, reported provocative results in the BRCA-mutated population (whilst naïve of PARP inhibitor) with an impressive 72% ORR (247). However, in the TOPACIO trial, a similar association combining pembrolizumab and niraparib, showed a lower ORR of 18% and 65% disease control rate in an heterogenous OC population. Interestingly, this association appeared particularly effective in the platinum-refractory and non-HRD populations (148). Finally, the DUO-O trial, which investigated the addition of durvalumab to olaparib and bevacizumab reported a significant improvement in outcomes in the BRCAwt/HRD population as expected with a PARPi in this context. However, similar to the TOPACIO trial, the BRCAwt/HRD-negative population also appeared to benefit from the combination even though PARPi alone have not shown effectiveness in this scenario (248). Altogether, these results suggest that combining PARP inhibitors with ICI may be a promising approach, especially in populations that are not usually candidate for PARP inhibitors. Nonetheless, the trial was not specifically designed to explore the benefits of the combination versus PARPi alone, and further dedicated trials are necessary to confirm these findings and identify the specific subset of patients that would derive the greatest benefit from this combination.

Overall, a multitude of ICI strategies are currently being explored in OC, and many of these trials consider an all-comer population. Translational studies to identify biomarkers that can predict response to ICI will be crucial in optimizing the use of these strategies in the future.

### 8.2 Monoclonal antibodies, cancer vaccines, and T-cell engagers

Cancer vaccines hold great promise in harnessing the patient’s own immune system to target tumor-associated antigens and induce specific immune responses against cancer cells. Several approaches have been tested in OC, showing encouraging activity in early trials. One strategy involves using cancer-testis/cancer-germline antigens such as NY-ESO-1, which are proteins aberrantly expressed in OC, and have demonstrated consistent CD4^+^ and CD8^+^ T-cell responses in OC patients leading to prolonged responses (253, 254). Another promising strategy involves DC vaccines, which capitalizes on the critical role of DCs in stimulating both adaptative and innate immunity. Schematically, DCs are collected from the patient’s blood, matured *in vitro* and then loaded with the specific antigen of interest. The matured, antigen loaded DCs are then injected back into the patient, stimulating a targeted immune response against the tumor. For example, vaccination with autologous DCs targeting Mucin 1 (MUC1), a glycoprotein highly expressed in ovarian carcinomas, resulted in a strong MUC1-specific T cell response and prolonged survival in patients with advanced OC (255). Similarly, a DC vaccine targeting folate receptor alpha (FRα) demonstrated a strong cytotoxic T-cell response (composed of IL-17 producing T cells) and prolonged remission in patients with heavily pretreated OC (256).

Additionally, monoclonal antibodies have been developed to mimic tumor antigens in order to induce a self-tumor antigen-specific response (anti-idiotypic antibody). Abagovomab was one such antibody imitating the tumor-associated antigen CA125. However. despite a sustained immune response and encouraging antitumor efficacy in early-phase trials, the drug failed to demonstrate a survival benefit in OC patients in later trials (257). The next-generation antibody oregovomab was designed to generate an immune complex with tumoral CA125, which is then processed by DCs and subsequently responsible for downstream CA125-specific antitumor immune response (258). Unfortunately, after an initial efficacy signal in a subset of OC patients with favorable prognostic characteristics, the phase III clinical trial did not show any statistical survival difference between oregovomab maintenance and placebo (259). Nonetheless, the FLORA-5 study (NCT04498117) is testing oregovomab in combination with chemotherapy in newly diagnosed OC and might demonstrate promising results.

Despite the promising results, and even if vaccine therapies are steadily moving forward, there are still multiple unresolved challenges. Manufacturing personalized vaccines for each patient can be time-consuming and costly, limiting their accessibility (260). In addition, as above-mentioned, DCs are highly plastic and can acquire an immunosuppressive phenotype over time, potentially limiting their long-term efficacy. Ongoing research is focused on developing more efficient and cost-effective methods of vaccine production and enhancing the stability and potency of dendritic cell vaccines. As research in this field continues to advance, cancer vaccines hold the potential to become an integral part of OC treatment, complementing other therapeutic modalities and improving patient outcome.

As previously mentioned, T-cell immune activation is mainly MHC restricted. Therefore, the impairment of antigen presentation by MHC molecules is an important immune evasion mechanism and a major limitation for T-cell activity (261, 262). T-cell engaging bispecific antibodies (bsAbs or BiTEs) are antibodies that have been designed to target both tumor-associated antigens and a T-cell molecule (most frequently CD3). This design directs polyclonal T-cells to the tumor resulting in tumor-specific cytotoxicity (263). As a result, bsAbs can crosslink cancer cells with T-cells independently of MHC action, limiting the potential for immune escape. Given the encouraging results in hematological malignancies, bsAbs are currently in clinical development for solid tumors. In OC, several tumors antigens are being tested including WT1 (264), MUC16 (265), claudin 6 (266) and folate receptor alpha (267). Notably, the toxicity profile of these therapies is of considerable concern due to the high rates of severe cytokine release syndromes (CRS) and immune effector cell-associated neurotoxicity (ICANS) (268, 269).

### 8.3 Adoptive cellular therapies

Adoptive cell therapy, including chimeric antigen receptor (CAR) T-cell therapy, is an innovative approach in cancer immunotherapy that involves the infusion of autologous immune cells that have been stimulated and expanded ex-vivo to target cancer cells. This approach aims to harness the patient’s preexisting antitumor response and enhance it, through the use of genetically modified immune cells.

The first trials, few decades ago, used TILS transfer in heavily pretreated diseases and showed encouraging activity and prolonged survival (270). More recently, the combination of ACT with PD-1/PD-L1 inhibitors has shown improved T-cell expansion and increased T-cell reactivity in OC patients, leading to promising antitumor activity even though it was tested only in a very limited number of patients (271).

Unlike autologous TILS infusion, CAR-T cells have been genetically engineered to target a specific tumor antigen, allowing an increased immune response with minimal off-tumor effect (272). Importantly, CAR-T cells are not MHC restricted and, as such, exert a highly efficient and specific tumor cytoxicity. In the context of OC, several potential antigens have been proposed as targets for CAR-T cell therapy due to their high expression levels and specificity. The most promising targets in OC are Mucin 16 (MUC16), mesothelin (MSLN), Folate receptor 1 (FOLR1) and tumor-associated glycoprotein 72 (TAG72) and have achieved promising responses both *in vitro* and *in vivo* (273–276). Clinical trials are actively recruiting patients with OC to test the safety and efficacy of these therapies (277). Finally, next-generation CAR cell therapies are also exploring the use of engineered NK cells, which are thought to limit systemic toxicity while enhancing the antitumor activity (278).

Although cellular immunotherapies come with important limitations including off-target toxicity and challenges in accessibility and manufacturing, they also hold great promise in OC treatment. Given the strong spontaneous antitumor response observed *in vivo*, they represent a potential breakthrough in the field of OC treatment.

## 9 Conclusions

In conclusion, despite preclinical data continue supporting the use of immunotherapy combinations, ICI is not yet firmly established in OC therapeutic landscape. This review aimed to summarize the potent immune evasion mechanisms deployed, the exceptional diversity of the immune cells and ligands recruited, presenting the OC TME both hindered by obstacles and a field of great opportunity. However, certain critical elements deeply intertwined into these pathways, such as extracellular matrix modeling, fibroblast recruitment, hypoxia, and metabolic reprogramming, as well as host factors, were intentionally overlooked in this work. These aspects gave been extensively covered by other works. For example, Yang et al., provided an extensive review of the immune and non-immune cellular components of the TME and how they can be targeted beyond immune-checkpoint blockade (279). Works by Kandalaft et al. and Colombo and colleagues, focused on the most promising strategies to target the TME considering the immunosuppressive context of OC and the potential future biomarkers (280, 281). Finally, Baci and colleagues reviewed the complex innate immune response in the OC TME and how it might either support or limit cancer progression and treatment sensitivity (282).

OC TME is a complex and dynamic landscape that plays a critical role in promoting tumor progression, invasion, dissemination, and treatment resistance. The immune cells and components within the TME possess both antitumor effects and immunosuppressive actions, creating a delicate balance that allows cancer cells to evade immune surveillance and continue to thrive. Understanding the interactions and polarization of these immune cells is essential for developing effective immunotherapeutic strategies. While there has been significant progress in understanding various features of the TME, such as extracellular matrix remodelling, cancer-associated fibroblasts, and metabolic reprogramming, there are still many unsolved enigmas and challenges to address. Cutting-edge techniques like single-cell sequencing and spatial molecular assays hold promise for unravelling the complexities of the TME and its interactions. In OC, it is likely to think that histological subtypes and molecular backgrounds be critical features to consider in the interpretation of results.

Immunotherapeutic approaches in ovarian cancer continue to show promise, and next-generation inhibitors, innovative combinations, vaccines, and cellular therapies offer exciting opportunities. However, finding the right target, tailoring therapies to individual patients’ immune characteristics, and carefully considering endpoints and potential toxicity will be critical for the success of these treatments.

## Author contributions

FB-D: Data curation, Writing – original draft, Writing – review & editing. LCWX: Data curation, Writing – review & editing. DT: Conceptualization, Supervision, Writing – review & editing.
